# Asymptomatic Survivors of Childhood Acute Lymphoblastic Leukemia Demonstrate a Biological Profile of Inflamm-Aging Early in Life

**DOI:** 10.3390/cancers14102522

**Published:** 2022-05-20

**Authors:** Eryk Latoch, Katarzyna Konończuk, Karolina Konstantynowicz-Nowicka, Katarzyna Muszyńska-Rosłan, Klaudia Sztolsztener, Adrian Chabowski, Maryna Krawczuk-Rybak

**Affiliations:** 1 Department of Pediatric Oncology and Hematology, Medical University of Bialystok, 15-274 Białystok, Poland; kononczukk@gmail.com (K.K.); kmroslan@post.pl (K.M.-R.); maryna.krawczuk-rybak@umb.edu.pl (M.K.-R.); 2 Department of Physiology, Medical University of Bialystok, 15-222 Białystok, Poland; karolina.konstantynowicz@umb.edu.pl (K.K.-N.); klaudia.sztolsztener@umb.edu.pl (K.S.); adrian@umb.edu.pl (A.C.)

**Keywords:** aging, cancer, CCS, childhood cancer survivors, children, chemokines, cytokines, growth factors, immunosenescence, inflammaging, inflammation, late effects, senescence

## Abstract

**Simple Summary:**

The most common malignancy diagnosed in childhood is acute lymphoblastic leukemia (ALL). With significant advances in ALL treatment and excellent supportive care, overall survival is now up to 90%, depending on the risk group. As a result, the survivor population is growing significantly. At the same time, it is observed that survivors experience many health problems that substantially reduce their quality of life. Emerging evidence suggests that the increased susceptibility to multiple disorders related to accelerated aging in CCS may be attributed to chronic inflammation. Therefore, this study aimed to investigate whether asymptomatic survivors of ALL have a biological phenotype of accelerated cellular senescence early in life. For this purpose, a broad panel of 51 cytokines was tested. We show that survivors after ALL treatment have an inflammatory profile associated with premature cellular aging. Factors that increase the risk of immunological alterations include younger age at diagnosis, high-risk protocols, and radiation therapy.

**Abstract:**

Childhood acute lymphoblastic leukemia (ALL) survivors are at higher risk of developing many late effects later in life. They experience multiple health problems that have significant public health implications, such as frailty, premature onset of lifestyle diseases, and second tumors. There is some evidence that chronic inflammation causes accelerated aging in childhood cancer survivors; however, the available data are very limited. The aim of the study was to evaluate the broad panel of cytokines among asymptomatic ALL survivors after anticancer treatment. The study included 56 subjects with a mean age of 16.11 ± 3.98 years. The commercially available Bio-Plex Pro Human Cytokine Screening 48-Plex Panel Assay and Bio-Plex TGF-β Assay were used for simultaneous determination of 48 cytokines and 3 isoforms of TGF-β. Among 51 tested cytokines, the levels of 33 were statistically significantly higher in ALL survivors than in the control group (*p* < 0.05). Increased levels of pro-inflammatory cytokines, including the IL-1 family (IL-1 β, IL-1Ra; *p* < 0.0001), IL-6 (*p* < 0.001), IL-17 (*p* < 0.001), IL-18 (*p* < 0.05), TNFα (*p* < 0.01), IFNα2 (*p* < 0.05), and IFNγ (*p* < 0.01), were found elevated in the entire study group, compared with the controls. Subjects treated previously according to the high-risk protocol had higher IL-18 levels than low- and intermediate-risk groups (*p* < 0.05). Elevated levels of IL-1ra, IL-6, IL-12 (p70), IL-17, LIF, M-CSF, CSF, and VEGF were found in ALL survivors treated before the age of 5, compared with subjects treated over 5 years of age (*p* < 0.05). Moreover, individuals who received radiotherapy presented elevated levels of both IL-18 (*p* < 0.05) and MIG (*p* < 0.05). In conclusion, we found that young asymptomatic survivors after ALL treatment demonstrated a biological profile of complex low-grade chronic inflammation.

## 1. Introduction

Acute lymphoblastic leukemia (ALL) is the most common malignancy in children. Currently, the overall long-term survival rate in children treated for ALL is approximately 80% due to advanced treatment and excellent supportive care [1,2]. Over the last five decades, there has been a significant increase in the number of individuals who have undergone treatment for childhood cancer. Recent large-scale studies have shown that these individuals experience various health problems more frequently and at a younger age than the general population [3,4]. Treatment-related secondary diseases include cardiovascular disease, metabolic syndrome, osteoporosis, neurocognitive disorders, infertility, and earlier mortality. Data from the literature indicate that more than 2/3 of survivors had at least one chronic condition, and almost 30% developed severe or even life-threatening sequelae [5]. Thus, anticancer treatment administered in childhood may cause individual adverse health effects later in life, leading to health inequalities and consequently may shorten lifespan. As a result, significant public health issues arise, including financial toxicity associated with managing many of the late sequelae.

One hypothesis holds that the differences in health status between childhood cancer survivors (CCS) and their equals result from premature cellular aging. Furthermore, emerging evidence suggests that the increased susceptibility to multiple disorders related to accelerated aging in CCS may be attributed to chronic inflammation [6]. Despite the described clinical phenotype of cellular senescence among CCS, the underlying pathogenesis at the intracellular level remains poorly explained. The available data are very limited, and the single studies conducted so far have only focused on single factors.

Anticancer therapy may lead to treatment-induced accelerated aging in several possible ways. They include cellular senescence, oxidative stress, telomere attrition, DNA damage, stem cell exhaustion, mitochondrial dysfunction, epigenetic alterations, etc. In general, cellular senescence is defined as the loss of the cells’ ability to proliferate properly, resulting in an irreversible arrest of cell growth [7,8]. This process is accompanied by an abnormal immune response—senescence-associated secretory phenotype (SASP), which is characterized by the release of multiple proinflammatory cytokines—interleukins (IL), chemokines, and growth factors. The SASP has been found to alter multiple metabolic pathways, promote inflammation, and tumorigenesis, and impair repair mechanisms, ultimately leading to accelerated cellular aging in CCS. The concept of inflamm-aging implies that low-grade inflammatory pathways observed in cancer survivors contribute to the loss of physiological capacity, resulting in impaired tissue and organ function [9]. However, multimodal treatment (high-dose chemotherapy, radiotherapy, biological therapy, etc.) is not indifferent to healthy tissues and nonmalignant cells [10].

In recent years, there has been an increase in the number of publications on the role of premature aging in patients with CCS; however, only few have examined the contribution of cytokines, and they have been limited to only few single proinflammatory cytokines (e.g., IL-1, IL-6, IL-17, TNF) [11]. Moreover, most studies have been conducted on adult CCS. It is highly plausible that the phenomenon of accelerated cellular senescence is multifactorial, with a far greater number of biomarkers involved, and may occur as early as childhood preceding the onset of many diseases in adulthood.

The aim of this study was to investigate whether asymptomatic children and young adult survivors of acute lymphoblastic leukemia have a biological phenotype of accelerated cellular senescence early in life. For this purpose, a broad panel of 51 cytokines was tested.

## 2. Materials and Methods

### 2.1. Study Population

**Figure 1:**
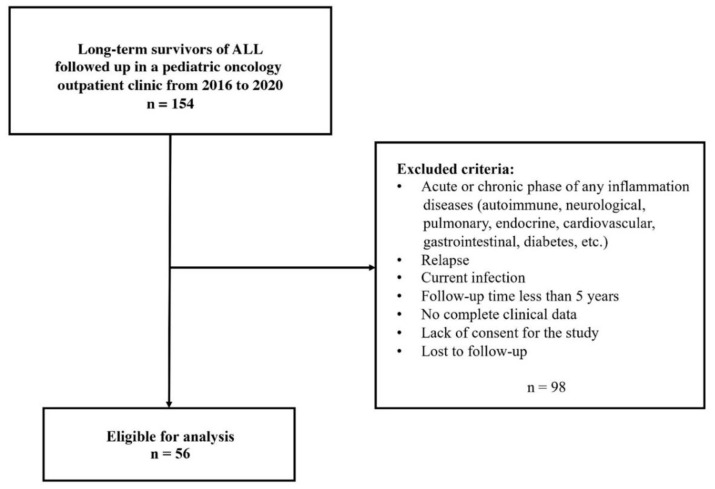
Flowchart presenting the selected study population.

Fifty-six White survivors of acute lymphoblastic leukemia, without clinical features of any comorbid disease, were recruited for the study. They gave written informed consent prior to the enrollment. The characteristics of the survivors’ cohort are presented in Table 1. The protocol of the International Berlin–Frankfurt–Münster Group (I-BFM) approved by the Polish Pediatric Leukemia and Lymphoma Group was used for treatment in childhood. Since the same protocol includes treatments of different intensities, the study group was divided into three subgroups according to the risk group (low, intermediate, and high risk). As the risk group increases, higher cumulative doses of cytostatic agents are used. In addition, high-risk protocols also consist of central nervous system (CNS) radiotherapy and/or hematopoietic stem cell transplantation (HSCT) in selected subgroups of children. Inclusion criteria included time of follow-up at least 5 years after the end of treatment, continuous first remission, and no comorbidities. Exclusion criteria are shown in Figure 1. The reference group consisted of 20 age- and sex-matched, cancer-free, healthy peers recruited from the children of hospital employees. All participants were carefully examined for any abnormalities on physical examination and the presence of inflammation using standard techniques. Medical history was taken rigorously. Data on age, sex, and anticancer treatment were obtained from medical records. Body mass index was calculated as weight divided by height (kg/m^2^). The waist-to-height ratio (WHR) was calculated by dividing waist circumference by height. Blood pressure was measured using a standardized sphygmomanometer. Hypertension (HT) was defined as a mean value of systolic blood pressure (SBP) and/or diastolic blood pressure (DBP) level ≥ 95th percentile adjusted for age, sex, and height. Echocardiography was performed to assess the heart’s structures and fraction shortening and ejection fraction by a pediatric cardiology specialist. The study was conducted in accordance with the Declaration of Helsinki and was approved by the local ethics committee (Permission Number: R-I-002/328/2019).

### 2.2. Multiplex Immunoassay

The commercially available Bio-Plex Pro Human Cytokine Screening 48-Plex Panel Assay (Catalog Number 12007283, Bio-Rad Laboratories, Hercules, CA, USA) and Bio-Plex TGF-β Assay (Catalog Number 171W4001M, Bio-Rad Laboratories, Hercules, CA, USA) allowed the simultaneous determination of 48 cytokines, as well as 3 isoforms of TGF-β (TGF-β1, TGF-β2, and TGF-β3) in each well of a 96-well plate, respectively. The Bio-Plex assays were performed on serum specimens according to the manufacturers’ instructions. The assay principle was based on the reaction with an antibody against a specific protein, which was covalently bound to fluorescently dyed magnetic beads, each with a distinct wavelength unique to the target protein. The bead-conjugated antibodies react with the sample containing the protein of interest. After the series of washes, biotinylated antibodies optional for different epitopes of the target proteins were added to the reaction. The final complex was made by adding a streptavidin–phycoerythrin (SA-PE) conjugate. A dual-laser, flow-based microplate reader—Bio-Plex 200 Reader (Bio-Rad Laboratories, Hercules, CA, USA)—detected the internal fluorescence of the individually dyed beads and the intensity of the signal on their surface. The obtained signal was expressed as median fluorescence intensity (MFI), analyzed, and presented as concentration (pg/mL) using the Bio-Plex Manager Software (Bio-Rad Laboratories, Hercules, CA, USA). The concentration of the analyte attached to the individual beads was proportional to the MFI of the phycoerythrin signal.

### 2.3. Statistical Analysis

The statistical analysis was performed using GraphPad Prism version 9.3.1 (GraphPad Software, San Diego, CA, USA). All values are expressed as a mean ± standard deviation (SD) or median (Me) and interquartile range (IQR) when appropriate. In the univariate analysis, Fisher’s exact test and χ^2^ test were used. For independent variables, the Mann–Whitney U test or Student’s *t*-test were used. We compared risk groups (standard, intermediate, and high) using univariate analysis of variance (ANOVA), and Tukey’s test was performed for post hoc analysis. Correlations were calculated by Spearman’s test. A statistical significance was determined at 0.05.

## 3. Results

The characteristics of the study group are shown in Table 1. The median age at diagnosis was 4.66 (2.62–7.40) years, and the follow-up time after treatment was 8.93 (7.06–11.81) years. The gender (*p* = 0.6) and median age of the study group did not differ from the control group—16.36 (13.32–19.27) years vs. 16.12 (14.16–17.65) years, *p* = 0.915.

**Figure 2:**
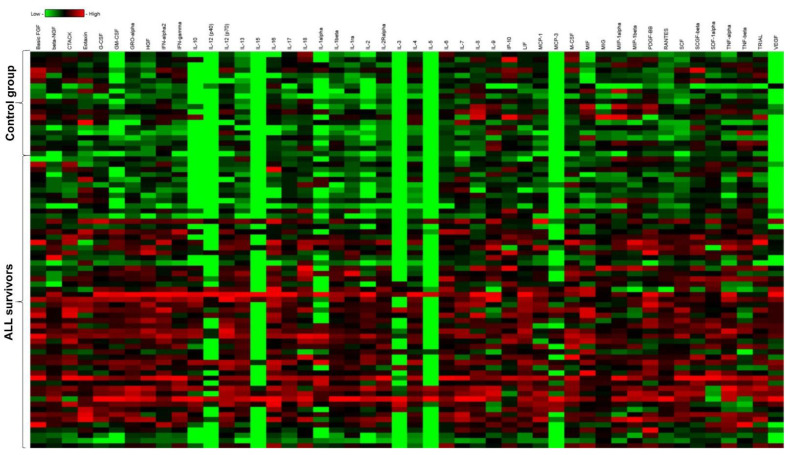
Heat map of cytokine levels in acute lymphoblastic leukemia survivors and controls.

Among 51 tested cytokines, the levels of 33 were statistically significantly higher in ALL survivors than in the control group (*p* < 0.05). A comparison of the cytokine profile in the study and reference groups is presented in Table 2 and Figure 2. The assessment of the levels of 5 out of 51 failed for technical reasons (IL-3, IL-5, IL-12(p40), IL-15, MCP-3).

Increased levels of proinflammatory cytokines—namely, the IL-1 family (IL-1 β, IL-1Ra; *p* < 0.0001), IL-6 (*p* < 0.001), IL-17 (*p* < 0.001), IL-18 (*p* < 0.05), TNFα (*p* < 0.01), IFNα2 (*p* < 0.05), and IFNγ (*p* < 0.01)—were found elevated in the entire study group [12].

**Figure 3:**
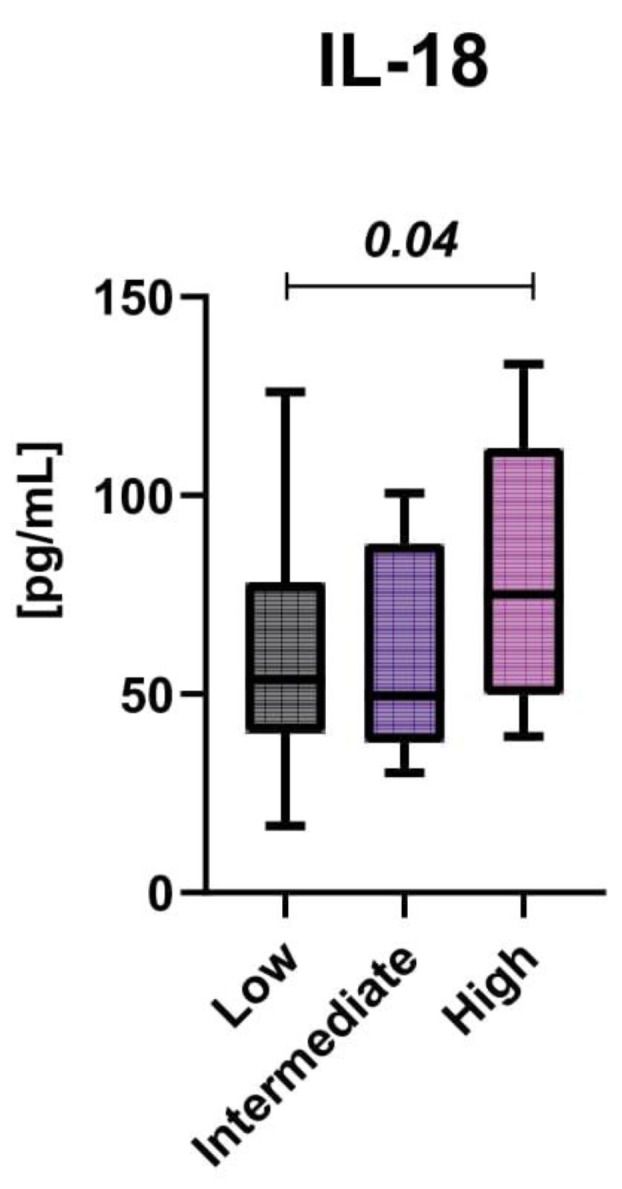
The mean interleukin 18 levels according to the treatment risk groups.

To determine the effect of treatment intensity on cytokine levels, survivors were divided into three groups according to the treatment protocols used in childhood. As a result, subjects treated previously according to the high-risk protocol had only higher IL-18 levels, compared with low- and intermediate-risk groups (*p* < 0.05), as shown in Figure 3. No differences were found in the concentrations of other cytokines between the analyzed subgroups.

**Figure 4:**
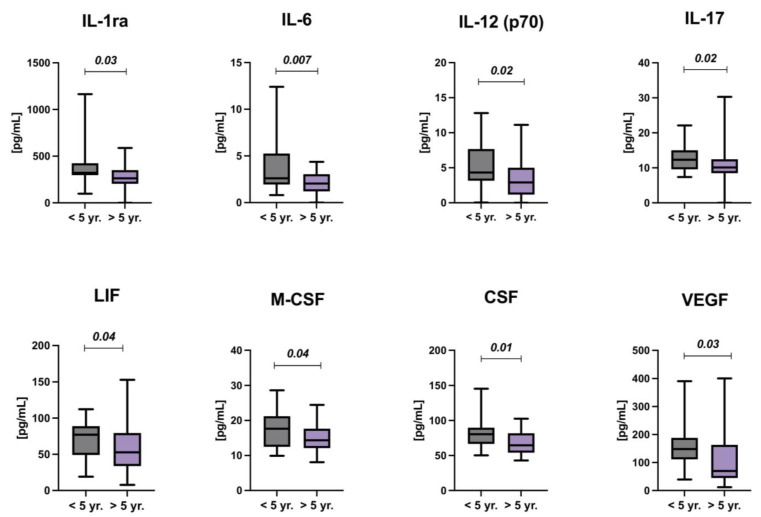
Levels of selected cytokines and chemokines by age at diagnosis (under and over 5 years of age).

Early childhood is a period of the rapid growth of the child and the development and maturation of many organs; hence, the treatment during this period may have an adverse effect on the later organ function. Therefore, the study group was also stratified by age at diagnosis—under and over 5 years of age. Increased levels of IL-1ra, IL-6, IL-12(p70), IL-17, LIF, M-CSF, CSF, and VEGF were found in ALL survivors treated before the age of 5, compared with subjects treated over 5 years of age (*p* < 0.05) (Figure 4).

The potential impacts of some cytostatic agents (anthracyclines, methotrexate, cyclophosphamide, and steroids) on serum cytokines and chemokines levels were further investigated; however, no effect was found.

**Figure 5:**
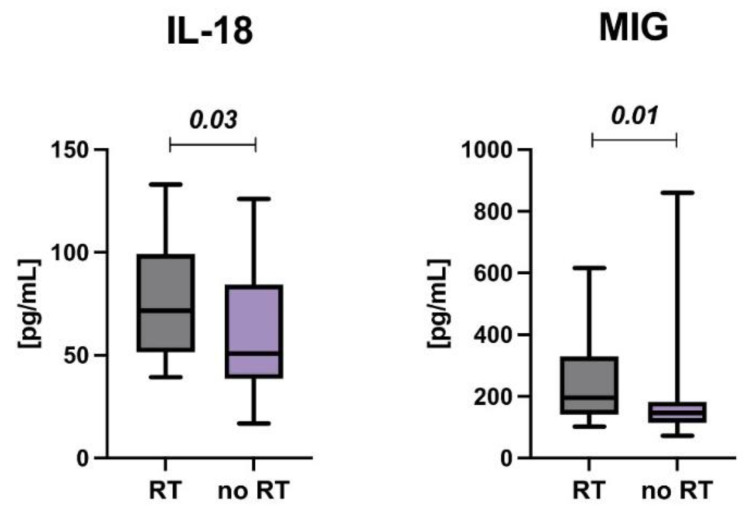
The interleukin 18 and monokine induced by gamma interferon (MIG) levels in childhood acute lymphoblastic leukemia survivors according to radiotherapy (RT) status.

One of the most harmful types of anticancer treatment, which is currently only targeted at carefully selected patients, is radiation therapy. In our cohort study, individuals who received radiotherapy had elevated levels of IL-18 (*p* = 0.037) and MIG (*p* = 0.012), as shown in Figure 5.

## 4. Discussion

Advances in acute lymphoblastic leukemia treatment have resulted in a significant increase in the number of survivors who live to adulthood. This population experiences many health problems much more frequently than their siblings or the general population [5,13]. In most cases, they develop civilization diseases, although long-term sequelae may affect any system or organ. This is attributed to a variety of treatment-dependent changes at the cellular level that most likely lead to accelerated senescence of CCS. Therefore, the aim of the present study was to investigate the pathophysiological basis for premature cellular aging as early as childhood and young adulthood by analyzing a broad panel of cytokines as a part of the senescence-associated secretory phenotype [14].

This cross-sectional study reported statistically higher levels of 33 cytokines and chemokines in ALL survivors than in the control group. Most of the available papers investigated a limited number of cytokines, chemokines, or growth factors. Ariffin et al. evaluated the levels of cytokines produced by helper T lymphocytes (Th) such as Th1 (IL-2, TNFα, IFNγ), Th2 (IL-4, IL-6, IL-10), and Th17 (IL-17A) in 87 asymptomatic ALL survivors. They showed a significantly higher concentration of IL-2, IL-10, and IL-17a, compared with controls [15]. Our study supports this finding; however, the study group also demonstrated higher levels of other tested cytokines such as TNFα, IFNγ, IL-6, and IL-17, except for IL-4. While the role of the best-studied proinflammatory cytokines is well-known, the high level of IL-17 in the context of its pleiotropic effects may be of interest. Interleukin 17 is secreted mainly by Th17, which under natural conditions activates neutrophils to inhibit bacterial and fungal pathogens. There is growing evidence, however, that it may also play a crucial role in the pathogenesis of chronic inflammatory diseases, adipogenesis, and cancer development by induction of other inflammatory cytokines, chemokines, and metalloproteases [16,17].

In contrast, in a study by Sulicka-Grodzicka et al. of 50 young adults with CCS, higher levels of IL-6 were not confirmed. It is noteworthy that in addition to higher levels of C-reactive protein and fibrinogen, they found a shift toward memory and activated T cells, higher activation of cluster differentiation marker (CD) 38, and a decrease in naive T lymphocytes [18]. Moreover, Bahri et al. showed that the CD38+ T cell might regulate immune activation during inflammation [19]. Although we did not analyze lymphocyte subpopulations in our study, the elevated levels of many cytokines produced by T cells in the study group may provide indirect evidence of enhanced T-cell activation in CCS.

Interleukin 18 (IL-18) is a member of the interleukin 1 (IL-1) family expressed by a range of inflammatory cell types. In our study, a higher level of IL-18 was found in high-risk patients than in those who were treated according to intermediate- or low-risk protocols. In addition, its higher levels were found in the subset of patients who were treated with radiation therapy compared with those in whom it was not used. IL-18, together with IL-12, induces high levels of IFNγ production by T cells, and its expression correlates with disease activities of rheumatoid arthritis and Crohn’s disease. Furthermore, in animal models, IL-18-deficient mice were more susceptible to bacterial infections than normal ones and showed uncontrolled disease progression. The assembly of the inflammasome in cells results in caspase-1 activation, followed by proteolysis and release of the cytokines IL-1b and IL-18 to induce pyroptotic cell death [11]. All these findings suggest that patients who were highly exposed to harmful treatments in childhood (more intensive chemotherapy and/or radiotherapy) have enhanced IL-18 production by activated macrophages, and thus a boosting effect of IL-18 on other immune cells.

Further stratification of patients in this study was based on age at diagnosis. The rationale for this approach was the hypothesis that children treated at a younger age are more likely to develop chronic inflammation due to the immaturity of many organs and systems. As expected, high levels of several cytokines and growth factors among ALL survivors treated before the age of 5 were found. They included IL-1 receptor antagonist (IL-1Ra), IL-6, IL-12(p70), IL-17, leukemia inhibitor factor (LIF), macrophage colony-stimulating factor (M-CSF), stem cell factor (SCF), and vascular endothelial factor (VEGF).

Unlike most cytokines of the IL-1 family, IL-1Ra has antagonistic effects on IL-1 α and β, which have exquisite proinflammatory potency. It binds competitively to the same receptor as IL-1 but does not transmit a cellular signal, thus inhibiting IL-1-induced cellular changes [20]. Contrary to our expectation, survivors of ALL who were treated at a younger age had significantly higher levels of IL-1Ra than older subjects. One of the explanations for that phenomenon may be the fact that the balance between expression levels of the IL-1 family, as well as activation and inhibitions of inflammasomes, and their receptors are critical in generating proinflammatory and homeostatic immune function [11,21,22]. Therefore, a compensatory upregulation of IL-1Ra in response to an increase in other IL-1 family cytokines cannot be excluded, although we did not observe this in our study.

Interestingly, IL-12(p70) expression was also higher in a group of younger children at the time of diagnosis. Their bioactive form IL-12(p70) is produced mainly by macrophages, monocytes, neutrophils, and dendritic cells, and it primarily mediates Th1 cell differentiation and maintenance. Moreover, it indirectly exerts an anti-infective function by activating the cytolytic activity of natural killer (NK) cells and Th1 cells [23]. On the other hand, data from the literature indicated that IL-12 may play an adverse role in inflammatory and autoimmune diseases, such as the central nervous system autoimmune diseases, uveitis, and multiple sclerosis. IL-12 induces naive CD4+ T cells to differentiate into Th1 cells, a subset of T-helper cells involved in the etiology of many autoimmune diseases in humans [24].

Leukemia inhibitory factor (LIF) is a member of the IL-6 family and is expressed in almost all types of body tissues. LIF was first discovered as a cytokine that induces blast differentiation in myeloid leukemia; however, studies of LIF in other diseases, including cancer, indicate that it may potentially contribute to many other pathologies. The role of LIF appears to vary greatly depending on the tissue in which it is expressed. LIF is involved in tumor spread and metastasis, treatment resistance, and cachexia, and has also cancer biomarker potential [25]. Since there are no studies available on cancer survivors, it is challenging to conclusively answer the question of the relationship between age of treatment and LIF expression and what role it may play in the morbidity of different diseases among CCS.

Macrophages play a crucial role in inflammation and tissue regeneration, and their differentiation is mainly regulated by M-CSF derived from different tissues [26]. Elevated levels of circulating M-CSF have been reported in multiple diseases including cancer, inflammation, and autoimmune disorders [27]. In contrast, VEGF is a well-known mediator of inflammation and is involved in tumorigenesis by regulating blood vessel permeability [28]. Finally, SCF is a cytokine that may contribute to the inflammatory changes that occur in diseases associated with increased numbers and activation of mast cells, such as asthma, etc. [29]. It could not be ruled out that elevated levels of growth factors among children treated at a younger age may be related to the earlier development of many diseases in CCS.

Increased levels of anti-inflammatory cytokines such as IL-4, IL-10, and IL-13 were also found in this study. Interleukin 4 is a major stimulant of Th2 cells and induces IgE production by B cells. Hence, it is involved in the development of allergy and is considered an important mediator of allergic inflammation [30]. In turn, IL-10 is a key anti-inflammatory mediator produced mainly by monocytes and T and B cells. It inhibits the expression of many proinflammatory factors and their receptors, providing host protection against overreaction to pathogens and microbiota [11]. IL-13 activates the same signal transduction pathways as IL-4. It acts through IL-13Ra1 and IL-13Ra2 receptors to induce IgE production [31]. A possible explanation for the elevated levels of some anti-inflammatory cytokines in ALL survivors may be an ongoing simultaneous process that leads to inhibition of inflammation and prevention of tissue damage due to chronic inflammation.

Moreover, it is important to note that ALL survivors showed elevated levels of regulated on activation, normal T-cell expressed and secreted (RANTES), which is a member of the CC chemokine subfamily and plays a chemotactic role for monocytes, T cells, and eosinophils. It may have deleterious effects by recruiting immune cells that enhance inflammatory diseases such as arteriosclerosis, arthritis, nephritis, dermatitis, colitis, and many other disorders [32]. There is also some evidence that RANTES may be involved in the thrombo-inflammation leading to various diseases [33,34].

Aside from premature death, the most serious late effects of cancer treatment in children are second malignancies occurring later in life. The link between SASP factors and tumorigenesis has remained one of the most engaging scientific issues for years. There is ample evidence to support this linkage. It is emphasized that SASP promotes tumor cell proliferation, dissemination, angiogenesis, and invasiveness [7,35,36]. Although anticancer treatment is targeted at destroying cancer cells, it also damages simultaneously normal cells, which release SASP factors that can paradoxically lead to the development of subsequent cancers among CCS later on.

In addition to cellular senescence induced by SASP factors, many potential mechanisms lead to premature aging that should be noted. These include DNA damage, stem-cell depletion, mitochondrial dysfunction, oxidative stress, telomere attrition, and epigenetic changes. Much effort is currently being made in identifying a group of measurements of accelerated aging in cancer survivors. Researchers implemented the concept of biological age to estimate accelerated aging in children with cancer to better predict the aging process than chronological age [37]. The risk of chronic and life-threatening conditions among CCS (e.g., frailty, organ dysfunction, civilization diseases, cognitive impairment, or secondary cancers) is expected to better predict biological age than chronological age. For this reason, the search continues for suitable markers with the greatest predictive value for the occurrence of treatment-related premature disorders [4,26,38]. A discussion of all the proposed biomarkers is well beyond the scope of this discussion. However, according to Wang et al., the most important measurements should include the epigenetic clock, proteomic aging clock, and two critical biomarkers of cellular senescence: p16^INK4a^ and alternate reading frame protein product of the CDKN2A locus (ARF) [15,39,40].

Finally, there are a few limitations to note. Firstly, single-center research should be interpreted in the context of the potential bias that may arise. Secondly, this pilot study was conducted on a relatively small group of individuals, which may affect the results. Finally, only cytokine levels as part of SASP were investigated, and no other parameters related to premature aging were analyzed. However, it is worth emphasizing that cellular senescence is a very complex process, and it is not possible to account for all factors and their interrelationships in a study performed on a small cohort of patients in one minor center.

Strengths of the study include a homogenous group of acute lymphoblastic leukemia survivors, a relatively long follow-up time, and no ethnic diversity. To the best of our knowledge, this is the first study to address such a broad panel of inflammatory biomarkers among childhood acute lymphoblastic leukemia survivors.

In conclusion, we demonstrated that ALL survivors present a biological profile of premature aging, manifested by increased secretion of multiple SASP biomarkers in the form of chronic inflammation. In addition, children treated for cancer at a younger age had higher levels of selected cytokines and growth factors than children treated at an older age, which may suggest a greater susceptibility of their healthy tissues to anticancer treatment administered in childhood. Furthermore, it is not yet clear how activation of cellular senescence mechanisms affects the development of different diseases. Additionally, in light of available studies indicating high morbidity among cancer survivors from multiple diseases, it seems reasonable to determine the exact pathogenesis of this condition. Thus, further longitudinal studies considering the contribution of a wide range of factors are needed.

## 5. Conclusions

In conclusion, we demonstrated that ALL survivors present a biological profile of premature aging, manifested by increased secretion of multiple SASP biomarkers in the form of chronic inflammation. In addition, children treated for cancer at a younger age had higher levels of selected cytokines and growth factors than children treated at an older age, which may suggest a greater susceptibility of their healthy tissues to anticancer treatment administered in childhood. Furthermore, it is not yet clear how activation of cellular senescence mechanisms affects the development of different diseases. Additionally, in light of available studies indicating high morbidity among cancer survivors from multiple diseases, it seems reasonable to determine the exact pathogenesis of this condition. Thus, further longitudinal studies considering the contribution of a wide range of factors are needed.

## Figures and Tables

**Table 1:** Clinical characteristics of the survivors of acute lymphoblastic leukemia (ALL).

	ALL Survivors
Total	56
Female	33
Male	23
Age at diagnosis (years)	5.65 ± 3.67 4.66 (2.62–7.40)
Age at study (years)	16.11 ± 3.98 16.36 (13.32–19.27)
Follow-up after treatment (years)	9.75 ± 3.63 8.93 (7.06–11.81)
**Risk groups**	
Standard	16 (29%)
Intermediate	27 (48%)
High	13 (23%)
**Chemotherapy ^a^**	
Glucocorticoids (cumulative dose in mg/m^2^ calculated as prednisone equivalents)	3600 ± 918.5 3087 (3087–3757)
Anthracyclines (cumulative dose in mg/m^2^)	225 ± 45 180 (180–240)
Cyclophosphamide (cumulative dose in mg/m^2^)	4086 ± 2628 3000 (3000–3375)
Methotrexate (cumulative dose in mg/m^2^)	10620 ± 7591 8000 (8000–8000)
**HSCT**	8 (14%)
**Radiotherapy**	12 (21%)
Cranial radiotherapy (CRT)	8 (14%)
Total body irradiation (TBI)	1 (2%)
CRT and TBI	3 (5%)
No radiotherapy	45 (80%)

ALL acute lymphoblastic leukemia, HSCT hematopoietic stem cell transplantation. Data are presented as mean ± standard deviation (SD) and median and interquartile range (IQR); categorical variables are presented as numbers (%). ^a^ Most patients received the same dosage of anticancer agents according to the treatment protocol; therefore, the first and third quartiles might not differ from the median.

**Table 2:** Cytokines profile in acute lymphoblastic leukemia (ALL) survivors and the reference group.

Cytokines (pg/mL)	ALL Survivors n = 56	Reference Group n = 20	*p*
TGF-β 1	129,043 (106,774–166,408)	122,118 (87,177–146,661)	0.447
TGF-β 2	2819 (2486–3169)	2763 (2530–2933)	0.571
TGF-β 3	550.2 (460.7–625.9)	540.5 (461.4–619.7)	0.878
CTACK/CCL27	961.2 (722.1–1343)	846.8 (718.8–942.7)	0.043
Eotaxin/CCL11	76.86 (51.52–94.74)	51.71 (41.13–74.77)	0.030
Basic FGF/FGF-2	58.83 (50.85–62.87)	58.83 (48.61–64.35)	0.491
G-CSF	87.52 (68.42–123.0)	74.49 (57.17–90.51)	0.021
GM-CSF	3.49 (1.65–5.44)	1.03 (0.69–1.80)	<0.001
GROα/CXCL1	372.6 (338.4–400.7)	310.9 (285.0–328.7)	<0.0001
HGF	459.0 (370.5–569.4)	297.2 (263.1–355.8)	<0.0001
IFN-α2	13.41 (10.32–15.64)	12.25 (9.31–12.95)	0.024
IFN-γ	13.58 (10.77–18.14)	9.72 (7.86–13.83)	0.003
IL-1 α	6.245 (2.71–9.76)	3.42 (2.71–6.24)	0.118
IL-1 β	3.6 (2.96–5.42)	2.375 (2.11–2.85)	<0.0001
IL-1 ra	311.2 (236.8–383.2)	191.1 (146.2–251.1)	<0.0001
IL-2	5.61 (3.36–7.37)	1.98 (1.02–4.16)	<0.001
IL-2R α	89.37 (69.90–102.1)	65.02 (60.66–77.80)	<0.001
IL-3	n/a	n/a	-
IL-4	3.36 (2.64–4.05)	3.00 (2.64–3.48)	0.163
IL-5	n/a	n/a	-
IL-6	2.44 (1.72–3.91)	1.15 (0.70–2.25)	<0.001
IL-7	22.97 (19.08–31.10)	17.08 (12.94–20.81)	<0.001
IL-8	8.60 (5.71–11.46)	6.30 (4.61–11.03)	0.247
IL-9	182.4 (167.5–205.2)	177.0 (160.0–195.0)	0.147
IL-10	1.19 (1.15–5.03)	0.14 (0.14–2.89)	0.036
IL-12 (p70)	4.09 (2.19–5.91)	1.94 (1.18–3.15)	0.002
IL-12 (p40)	n/a	n/a	-
IL-13	4.90 (1.92–6.97)	1.04 (0.88–2.06)	<0.0001
IL-15	n/a	n/a	-
IL-16	57.96 (28.63–80.68)	19.74 (15.04–28.77)	<0.001
IL-17	11.75 (9.158–13.93)	8.205 (6.97–10.66)	<0.001
IL-18	53.19 (40.37–87.97)	46.12 (37.69–54.35)	0.042
IP-10/CXCL10	380.3 (284.7–545.0)	353.3 (225.1–492.5)	0.348
LIF	65.08 (45.95–87.78)	45.32 (27.23–50.33)	0.002
MCP-1/CCL2	36.86 (24.40–57.11)	23.72 (18.56–28.42)	<0.001
MCP-3/CCL7	n/a	n/a	-
M-CSF	15.36 (12.47–19.63)	12.13 (11.11–13.82)	0.005
MIF	793.9 (536.0–1287)	631.4 (366.8–955.2)	0.046
MIG	153.8 (120.1–208.6)	112.2 (78.32–145.0)	0.001
MIP-1α/CCL3	2.05 (1.77–2.47)	1.795 (1.46–2.65)	0.235
MIP-1 β/CCL4	99.6- (90.68–112.1)	94.60 (84.26–107.0)	0.078
beta-NGF	1.06 (0.52–1.635)	0.800 (0.362–0.800)	0.019
PDGF-BB	4326 (3159–5675)	3053 (2363–4356)	0.008
RANTES/CCL5	30,543 (19,065–39,843)	14,917 (13,099–18,121)	<0.0001
SCF	75.93 (60.0–86.99)	51.44 (42.70–63.42)	<0.0001
SCGF-β	139,740 (120,468–162,814)	126,740 (104,353–160,661)	0.129
SDF-1α	707.2 (634.8–809.7)	623.9 (572.9–678.1)	0.006
TNF-α	61.99 (50.1–72.25)	52.69 (42.29–57.86)	0.002
TNF-β	215.8 (194.7–236.3)	205.6 (195.8–227.0)	0.485
TRIAL	52.39 (44.76–62.46)	47.64 (37.70–54.38)	0.039
VEGF	141.2 (67.29–184.9)	33.28 (12.05–80.08)	0.008

n/a, not applicable; RANTES, regulated on activation, normal T-cell expressed and secreted. Data are given as the median (Me) with interquartile range (IQR).

## Data Availability

The data that support the findings of this study are available from the corresponding author upon reasonable request. Some data may not be made available because of privacy or ethical restrictions.
